# Modulation of gene expression in guinea pig paraflocculus after induction of hearing loss

**DOI:** 10.12688/f1000research.3594.2

**Published:** 2014-05-01

**Authors:** Wilhelmina H. A. M. Mulders, Jennifer Rodger, Clarissa G. Yates, Donald Robertson

**Affiliations:** 1 The Auditory Laboratory, School of Anatomy, Physiology and Human Biology, The University of Western Australia, Crawley, WA, WA6009, Australia; 2 School of Animal Biology, The University of Western Australia, Crawley, WA, WA 6009, Australia

## Abstract

Hearing loss often results in plastic changes in the central auditory pathways, which may be involved in the generation of tinnitus, a phantom auditory sensation. However, although animal studies have consistently shown increased neural activity in auditory structures after hearing loss, tinnitus does not always develop. It has therefore been suggested that non-auditory structures perform a gating or regulatory role that determines whether the increased activity in auditory structures leads to conscious perception. Recent evidence points to the paraflocculus of the cerebellum as having such a role. Therefore, we investigated the early effects of hearing loss on gene expression in guinea pig paraflocculus. Gene expression was investigated after two weeks recovery from either acoustic or mechanical cochlear trauma. The genes investigated in our study were associated with inhibitory neurotransmission (GABA-A receptor subunit alpha 1; glutamate decarboxylase 1), excitatory neurotransmission (glutamate receptor NMDA subunit 1), and regulation of transmitter release (member of RAB family of small GTPase). Our results show increased mRNA levels of glutamate decarboxylase 1 in ipsilateral paraflocculus with no difference between the different methods of cochlear trauma. Early modulation of gene expression in the paraflocculus suggests that an early effect of hearing loss may affect the influence of this structure on auditory processing.

## Introduction

It is well known that trauma to the cochlea results not only in a reduced sensitivity to sound ^1, 2^, but also leads to a variety of physiological changes in the central nervous system. Central neural changes following hearing loss have been described using many different animal models and include changes in tonotopic maps ^3, 4^, increased synchronous firing patterns ^5– 7^ and increased spontaneous firing rates ^6– 11^.

This abnormal neural activity observed in the central auditory pathways following cochlear trauma has been suggested to play a role in the development of tinnitus, a phantom auditory sensation that is often associated with the presence of hearing loss ^12– 14^. However, although animal studies have shown that central changes are consistently present after trauma to the cochlea, tinnitus is not always present ^15– 17^. This is in accordance with human data showing that not all subjects with hearing loss develop tinnitus ^18^.

Therefore, although a trauma to the peripheral auditory receptor could serve as a trigger for the abnormal changes seen in the central auditory pathways, other brain regions are likely to be involved in giving rise to the eventual phantom auditory perception. Some brain regions that have been suggested are limbic structures ^19– 21^, which may be involved in gating mechanisms for the suppression of unwanted noise. Another structure that may be involved in this process is the paraflocculus of the cerebellum, which is not considered part of the classical auditory pathway ^22^. Brozoski and co-workers reported that neural activity increases in the paraflocculus of rats displaying behavioural evidence of tinnitus ^23^. Furthermore, ablation of the paraflocculus in rats before the induction of tinnitus results in the prevention or reduction of subsequent tinnitus ^17^ and infusion of a NMDA antagonist into the paraflocculus of rats with tinnitus decreases both their tinnitus and the associated elevated activation in cochlear nucleus ^24^.

Interestingly, anatomical data suggest that the paraflocculus receives direct sensory inputs from the cochlea ^25^. This is in line with the observation that the paraflocculus neurons of rats ^26^ and bats ^27^ respond to auditory stimuli. In addition, Azizi *et al.* showed evidence of connectivity between central auditory structures and the paraflocculus. In particular, they showed that rat paraflocculus neurons can respond to electrical stimulation of the auditory cortex and inferior colliculus. Using anterograde and retrograde tracer techniques, the authors also showed evidence for a corticopontocerebellar connection from the auditory cortex to the paraflocculus ^28^.

In central auditory structures, hearing loss might result in transcriptional modulation of genes regulating inhibitory and excitatory neurotransmission, regulation of pre-synaptic transmitter release and intrinsic neuronal membrane excitability ^9, 29– 34^. In view of the direct cochlear input to the paraflocculus and the indirect innervation arising from the auditory cortex, gene expression changes might also be found in paraflocculus soon after hearing loss. We therefore investigated peripheral hearing loss and used quantitative real-time PCR (qRT-PCR) to measure the expression of four genes involved in inhibitory and excitatory neurotransmission and regulation of pre-synaptic transmitter release in paraflocculus of guinea pigs following acoustic and mechanical cochlear trauma. Both of these methods of trauma are known to result in hearing loss, hyperactivity and associated changes in gene expression in the central auditory pathway ^9, 10, 34^. We studied the expression of GABA-A receptor subunit alpha 1 (GABRA1), glutamate decarboxylase 1 (GAD1), glutamate receptor NMDA subunit 1 (GRIN1) and a member of Rab family of small GTPase (RAB3A) using guinea pig-specific primers previously designed in our laboratory ^9^.

## Methods

### Animals

Twelve (8 males and 4 females) adult pigmented guinea pigs (tricolor strain obtained from a breeding colony at the University of Western Australia) weighing between 245 and 385g at the time of final recovery surgery were used in the experiment. Animals were housed in cages with clear plastic walls (51 cm × 41 cm base, 28 cm high) based on sex (2 per cage) in a SPF facility at 22°C with a 12 hour light/dark cycle. Guinea pigs had ad libitum access to food and water. Animals were supplied with Perspex hutch and hay for environmental enrichment. We investigated the effects of three treatments (sham, acoustic trauma and mechanical trauma) on peripheral thresholds and measured gene expression in the paraflocculus by RT-PCR. Animals were randomly allocated to an experimental group (n=4 for each). All experimental protocols were in line with the Code of Practice of the National Health and Medical Research Council of Australia, and were approved by the Animal Ethics Committee of The University of Western Australia, approval ID 03/100/1007.

### Anaesthesia and surgery

Anaesthesia and surgical procedures have been described in detail previously ^9, 35^. For recovery experiments,all animals were injected with 0.1ml atropine sulphate (0.6 mg/ml; APEX Laboratories, Australia) subcutaneously, followed by an intraperitoneal injection of diazepam (5 mg/kg; Pamlin, Ceva, Australia), and an intramuscular injection of Hypnorm 20 minutes later (0.315 mg/ml fentanyl citrate and 10 mg/ml fluanisone; 1 ml/kg; VetaPharma, UK). Absence of the foot withdrawal reflex was used to ascertain deep anaesthesia, after which animals were placed on a heating blanket in a soundproof room and the head mounted in hollow ear bars. A small opening was made in the bulla on the left side and an insulated silver wire was placed on the round window in order to record a compound action potential (CAP) audiogram (frequency range 4–24 kHz in 2 kHz steps) ^36^ using a closed sound system. All sound stimuli were presented through a ½" condenser microphone driven in reverse as a speaker (Bruel and Kjaer, type 4134). Pure tone stimuli were synthesized by a computer equipped with DIGI 96 soundcard connected to an analog/digital interface (ADI-9 DS, RME Intelligent Audio Solution). Sample rate was 96 kHz. The interface was driven by a custom-made computer program (Neurosound, MI Lloyd), which was also used to collect single neuron data during the non-recovery experiment. CAP signals were amplified, filtered (100 Hz–3 kHz bandpass) and recorded with a second data acquisition system (Powerlab 4SP, AD Instruments). If cochlear thresholds were within the normal range, the animal was randomly assigned to either the sham group, acoustic trauma or mechanical lesion group.

Sham animals received no further treatment after the measurement of the CAP audiogram, but this group was treated identically to the acoustic trauma and mechanical trauma group in every other aspect; they were maintained under anaesthesia for two hours and underwent identical recovery treatment.

For acoustic trauma groups, the contralateral ear was blocked with plasticine and the animal was subjected to a continuous loud tone for 1 hour (10 kHz, 124 dB SPL). After the acoustic trauma, CAP thresholds were again recorded to determine the magnitude of the immediate hearing loss.

For mechanical lesions groups, a small hole was hand drilled in the wall of the cochlea at the level of the basal turn. A glass micropipette electrode (tip diameter ~20 μm) filled with 150 mM KCI was inserted through the hole passing through the scala tympani and the organ of Corti into the scala media (signalled by the sudden appearance of a large positive potential between 80 and 100 mV). The pipette was then further advanced until it penetrated Reissner’s membrane (signalled by a drop in the positive voltage). The pipette was then removed and a CAP audiogram was determined to establish loss of neural sensitivity. This procedure was repeated up to 3 times to ensure a substantial change in CAP thresholds, after which the hole in the cochlear wall was covered by a small piece of gelfilm.

All animals remained under full surgical anaesthesia throughout the acoustic and mechanical trauma procedures. Finally, in all animals the incision was sutured and buprenorphine (0.05 mg/kg subcutaneously; Temgesic, Reckitt Benckiser, Australia) was given post-operatively as analgesic. Survival time from the recovery experiment till the final non-recovery experiment was 2 weeks.

For the final non-recovery experiments anaesthesia consisted of a subcutaneous injection with 0.1ml atropine followed by an intraperitoneal injection of pentobarbitone sodium (30 mg/kg; Ilium, Australia) and an intramuscular injection of Hypnorm (0.15ml). Animals were placed on a heating blanket in a sound proof room and artificially ventilated on carbogen (95% O _2_ and 5% CO _2_). After the animals were mounted in hollow ear bars, the left and right cochleae were exposed and CAP audiograms were recorded on both sides with a silver wire placed on the round window as described above for the recovery experiments.

### Tissue collection

Following the measurements of the CAP audiograms during the non-recovery experiment, for tissue collection, animals were terminally anaesthetised with Pentobarbitone (Lethabarb, Virbac, Australia) and decapitated using an animal guillotine (World precision Instruments, USA) and their brains rapidly removed in ice-cold phosphate-buffered saline (0.1M). The paraflocculus, on both sides of the brain, was removed quickly using either a sharp scalpel or fine scissors, and then transferred into 1.5ml RNase-free tubes. The samples were immediately stored at −80ºC until RNA extraction.

### qRT-PCR

The qRT-PCR procedures have been described in detail previously ^29^. The total RNA was isolated using a tissue homogenizer (Invitrogen, Mount Waverley, VIC, Australia) and a PureLink RNA Mini Kit Total RNA Purification System (Invitrogen), according to the manufacturer’s protocol for purifying RNA from frozen animal tissue followed by a standard ethanol precipitation. Nucleic acid concentration was measured by a NanoDrop 1000 Spectrophotometer (Thermo Scientific, Waltham, MA, USA). Subsequently, genomic DNA contamination was removed by RQ1 RNase-free DNase (Promega, Alexandria, NSW, Australia) treatment (1U/µg nucleic acid). The RNA integrity was confirmed by agarose gel electrophoresis before storage at −80°C.

The following four genes were selected for qRT-PCR: the GABA-A receptor subunit alpha 1 (GABRA1), GAD1, GRIN1 and RAB3A. The guinea pig-specific primers for these genes were designed previously in our laboratory ^9^. Synthesis of first-strand cDNA from RNA was carried out using GoScript Reverse Transcription System (Promega) and 500 ng of RNA was reverse transcribed in a 20 µl reaction with random primers according to the manufacturer’s instructions. The resultant cDNA was purified in a PCR clean-up kit (Promega Wizard PCR clean-up system). The purified cDNA was quantified again on a Nanodrop 1000 Spectrophotometer and diluted 40 times with nuclease-free water before being stored at −80°C.

qRT-PCR was performed in a Rotor-Gene Q real-time thermocycler (Corbett Life Science, Sydney, NSW, Australia). Amplification was carried out in a total volume of 20 µl reaction mixture containing 10 µl of 2× QuantiTect SYBR Green PCR Master Mix (catalogue number: 204141; Qiagen, Doncaster, VIC, Australia), 0.5 µM of each specific gene primer and 9 µl (10 ng) of diluted cDNA prepared as described above. Real-time PCR reactions were cycled 40 times after initial denaturation (50°C for 2 min, 95°C for 15 min) under the following parameters: 94°C for 15 s (denaturation), 54°C for 30 s (annealing) and 72°C for 30 s (extension and fluorescence data collection). Samples were run in duplicate and accompanied by negative controls (‘no reverse transcription’ and ‘no template’). The specificity of all amplicons was further assessed by using the melting curve protocol on the Rotor-Gene Q (Corbett Life Science). In order to avoid problems created by any inter-run variability, qRT-PCR for tissue samples (controls, acoustic and mechanical trauma) from the same side of the brain was conducted in the same runs. All analyses were replicated for each gene and the mean of the two reactions was used to calculate the expression level of that gene in each animal. Using the housekeeping genes ribosomal protein S16 (RPS16) and beta-2-microglobulin (B2M) for normalization ^30^, relative quantification of target gene expression for all groups was performed following the comparative CT method ^37^. In order to reflect clearly the different expression levels of different genes, the data are reported only as the ratio of target to housekeeping gene without converting to fold change. To calculate the ratio of target to housekeeping genes, for each target gene the mean of the replicates was calculated as well as the mean of the replicates of both the housekeeping genes.

### Data analysis

Statistical analysis of CAP threshold changes following each treatment and at each frequency was performed using a Kruskall-Wallis test and a Dunn’s multiple comparison post-test. Statistical significance (estimated at p<0.05) for qRT-PCR data was evaluated using ANOVA with Bonferroni’s multiple comparison post-tests (GraphPad Prism software).

## Results

### Peripheral auditory thresholds

**Figure 1.**
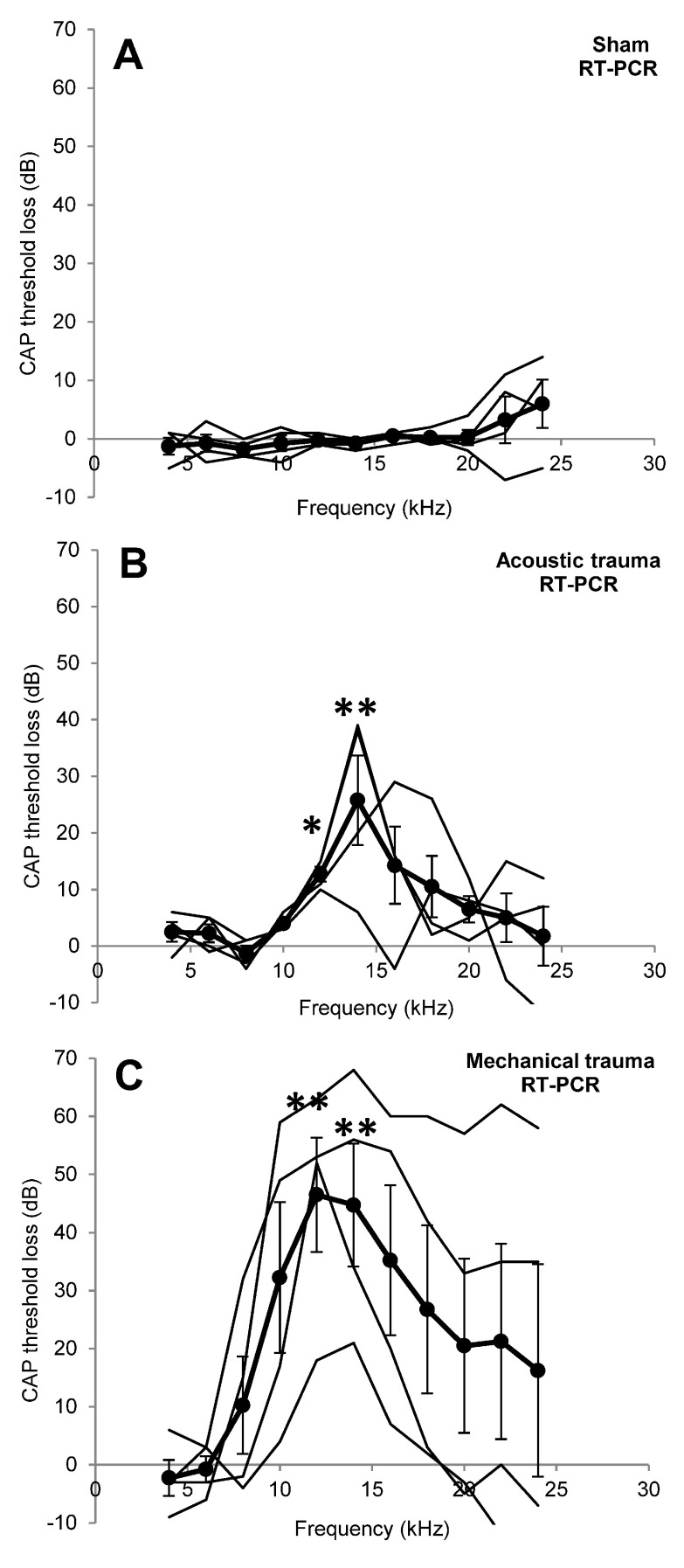
Peripheral hearing loss. Changes in cochlear sensitivity measured as CAP threshold loss recorded in the left cochlea after recovery from sham surgery ( **A**), acoustic trauma ( **B**) or mechanical trauma ( **C**). Thick black lines indicate the mean ± SEM (n=4 for all), thin black lines represent individual animals. * *p*<0.05, ** *p*<0.01 compared to before treatment data.

The effects of different treatments on CAP peripheral thresholds are illustrated in Figure 1. Sham surgery had no significant effect on peripheral thresholds at 2 weeks recovery ( Figure 1A). Acoustic and mechanical trauma both resulted in a frequency restricted hearing loss after recovery, though there was large variation in the patterns of hearing loss between the individual animals ( Figure 1B, C, thin black lines for individual animals). Statistical comparisons of the groups showed that there were significant threshold losses at 12 kHz and 14 kHz in both groups (acoustic trauma p<0.05 at 12 kHz and p<0.01 at 14 kHz; mechanical trauma p<0.01 at 12 and 14 kHz). There were no statistically significant differences in threshold loss between the acoustic and mechanical trauma group. Thresholds measured in the right ear were not significantly different from the left ear before trauma ( Data Set 1).

### Gene expression in the paraflocculus

**Figure 2.**
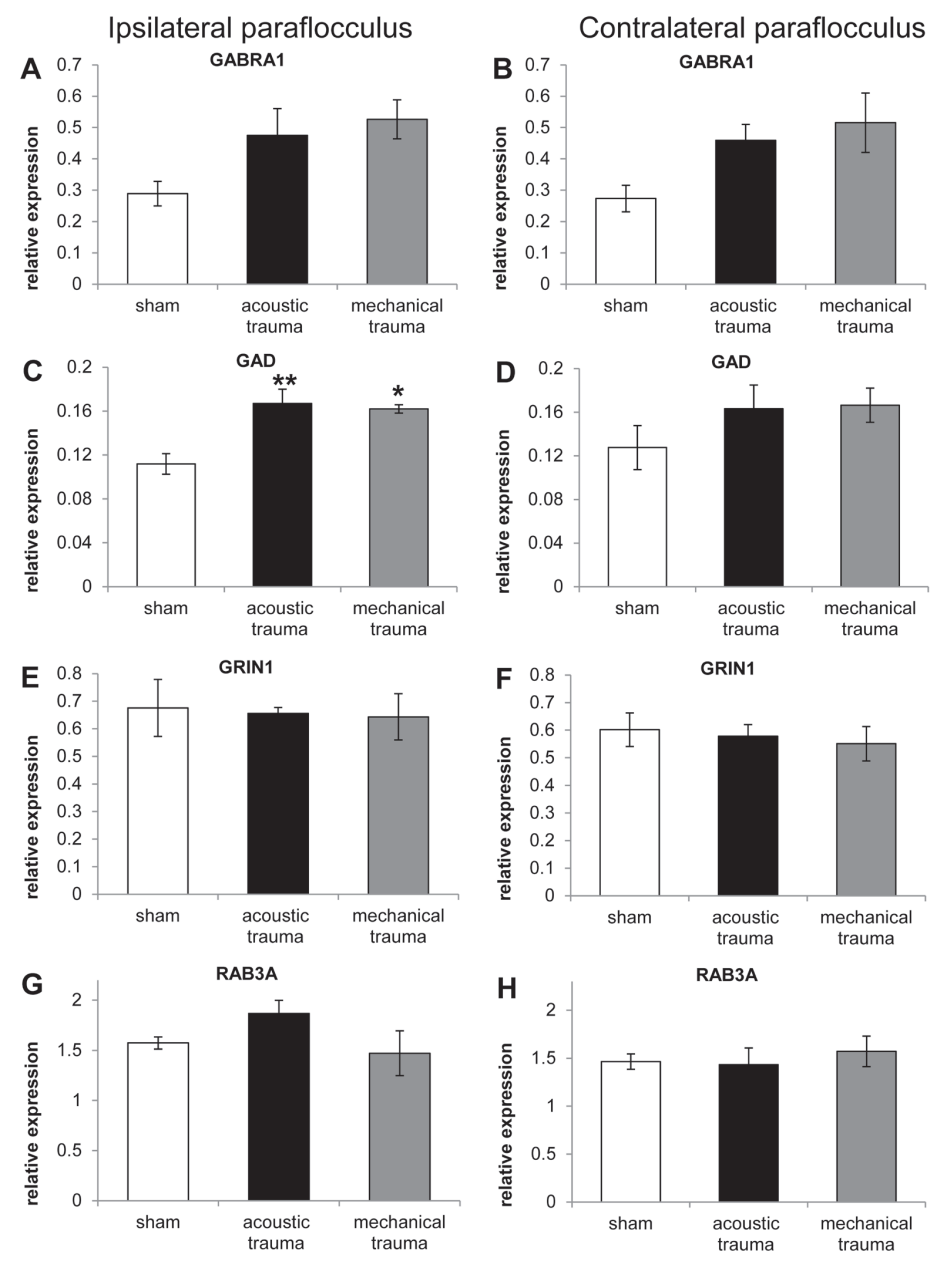
RT-PCR data from paraflocculus in animals subjected to sham, acoustic trauma and mechanical trauma. Changes in mRNA expression levels of 4 genes in the left (ipsilateral; **A**, **C**, **E**, **G**) and right (contralateral; **B**, **D**, **F**, **H**) paraflocculus in sham (white bars), acoustic trauma (black bars) and mechanical trauma animals (grey bars), after 2 weeks recovery, as shown by qRT-PCR. Gene abbreviations: GABR1: GABA-A receptor subunit alpha 1; GAD1: glutamate decarboxylase 1; GRIN1: glutamate receptor NMDA subunit 1; RAB3A: a member of RAB family of small GTPase. Values are mean ± SEM. Statistical analysis: * *p*<0.05, ** *p*<0.01. Asterisks indicate comparison with sham data.

Figure 2 shows the pattern of gene expression in the paraflocculus ipsilateral and contralateral to the cochlea exposed to sham surgery, acoustic trauma or mechanical trauma. No statistically significant changes were observed between the ipsi- and contralateral side in sham animals for any of the genes investigated. Two genes (GRIN1, involved in excitatory neurotransmission and RAB3A, involved in regulation of pre-synaptic neurotransmitter release Figure 2E–H) did not show any change compared to sham animals after either acoustic or mechanical trauma to the cochlea.

However, for the genes involved in inhibitory neurotransmission (GABRA1 and GAD1) there was an upward trend compared to sham animals following both acoustic and mechanical trauma ( Figure 2A–D). For GABRA1 the increase after acoustic and mechanical trauma compared to sham animals varied from 64% to 88% and for GAD1 increases varied from 27% to 49%. The percentage of increase was calculated as: (trauma value/sham value)/(sham value/100). ANOVA performed on the ratios of the target to housekeeping genes showed a significant effect of treatment only for GAD1 expression in the ipsilateral paraflocculus F(2, 9) = 10.19, p=0.0049 (ANOVA test). Bonferroni’s post-hoc analysis showed significant increases of 49% (p<0.01) and 45% (p<0.05) compared to the sham group after acoustic and mechanical trauma, respectively ( Figure 2C, D).

Data of paraflocculus cochlear trauma and modulation of gene expression. Cochlear threshold data Spreadsheet showing animal number (column A), treatment of each animal (column B) and cochlear threshold data. Cochlear threshold data are supplied as compound action potential (CAP) threshold values for frequency range 4 to 24 kHz in dB SPL determined ipsilaterally before treatment (Columns A-K), immediately after treatment (Columns L-V) and 2 weeks after treatment (Columns W-AG) as well as contralaterally 2 weeks after treatment (Columns AJ-AT).
RT-PCR Data Spreadsheet showing animal number (column A), treatment of each animal (column B) and replicate Ct values for gene of interest and for housekeeping genes ribosomal protein S16 (RPS16) and beta-2-microglobulin (B2M) (column E), from samples from left and right paraflocculus (PF). Columns C-H data for GABA-A receptor subunit alpha 1 (GABRA1); columns I-N data for glutamate decarboxylase 1 (GAD1); columns O-T data for glutamate receptor NMDA subunit 1 (GRIN1); columns U-Z data for a member of RAB family of small GTPase (RAB3A).

## Discussion

The present study shows the transcriptional modulation of genes regulating neurotransmission in the paraflocculus of the cerebellum of guinea pigs, following peripheral hearing loss due to damage of the cochlea. Changes in genetic expression are caused by either acoustic or mechanical trauma to the sense organ. These early changes in paraflocculus observed after two weeks may be evoked by an alteration in the direct input from the cochlea or in the indirect pathway described from the cortex as outlined below.

The hearing loss after acoustic trauma observed in our experiment showed a notch–like loss that was largest just above the exposure frequency, as reported in previous studies ^10, 29, 38– 40^. This pattern of hearing loss can be attributed to the nonlinear properties of the basilar membrane at high sound levels ^41, 42^. Mechanical trauma in this study also resulted in notch-like losses with the largest loss observed at the same frequency as after acoustic trauma.

Our RT-PCR analysis performed in the paraflocculus following mechanical or acoustic trauma of the cochlea revealed no significant changes in the expression of the glutamate receptor NMDA subunit GRIN1, associated with excitatory neurotransmission, or RAB3A, involved in regulation of neurotransmitter release ^43^. However, both types of trauma induced a slight increase in mRNA levels of genes associated with inhibitory neurotransmission (GABRA1 and GAD1), which was statistically significant for GAD1 in the ipsilateral paraflocculus. Changes in the ipsilateral paraflocculus may be a direct effect of an altered cochlear input to this cerebellar structure after trauma, since it has been demonstrated in chinchilla that following an injection with biotinylated dextran amine in the cochlea, labelled axons were found in the ipsilateral paraflocculus ^25^. In addition, paraflocculus neurons in rats ^26^ and bats ^27^ have been reported to respond to free field auditory stimuli. The apparent increase in gene expression in the contralateral paraflocculus, although not statistically significant, may be an indirect effect due to changes in neuronal activity in the inferior colliculus or cortex. Azizi and co-workers demonstrated that paraflocculus neurons respond to electrical stimulation of the auditory cortex and inferior colliculus and showed evidence for a corticopontocerebellar connection ^26, 28^.

Interestingly, changes to the ipsilateral rather than the contralateral paraflocculus are in line with the changes in the neural activity in paraflocculus of rats with tinnitus induced by acoustic trauma reported by Brozoski and co-workers ^23^. However, our finding of an increase in gene expression levels associated with inhibitory actions seems counter-intuitive in view of the increase in activity reported ^23^. However, Brozoski *et al.* tested their rats approximately 12 months after acoustic trauma, whereas the present study was performed two weeks after acoustic trauma. Other studies have shown that gene expression after acoustic trauma can vary significantly at different time-points ^29^ and the increase in inhibition observed in the present study may represent only an early transient change. In addition, depending on the circuitry affected by the altered gene expression, the downstream effect on the activity in the core auditory pathway may well be disinhibition and therefore it may cause an increase in physiological activity. It is still unclear whether there are direct efferent projections from the paraflocculus to the auditory system and whether these are excitatory or inhibitory.

Our data demonstrate early changes in the paraflocculus following two different types of cochlear trauma that both result in hearing loss and hyperactivity in the central auditory nuclei ^9, 38^. At present, the functional consequences of these changes in gene expression, in particular for tinnitus related activity in the auditory pathway, remain to be determined.

## Data availability

*figshare*: Data of paraflocculus cochlear trauma and modulation of gene expression. http://dx.doi.org/10.6084/m9.figshare.938193 ^44^.
