# Supplementary material for: Modulation of gene expression in guinea pig paraflocculus after induction of hearing loss
Source: F1000Res. 2014 May 1;3:63. Originally published 2014 Feb 27. [Version 2] doi: 10.12688/f1000research.3594.2 (PMC4207248; doi:10.12688/f1000research.3594.2)
Supplement: Peer review history

# Peer review: Referee response for version 2

**DOI:** 10.5256/f1000research.4325.r4619

**Published:** 2014-05-27

**Authors:** Ramesh Rajan

**Affiliations:** 1 Department of Physiology, Monash University, Sydney, Australia

I approve the article with the changes made by the authors.

I have read this submission. I believe that I have an appropriate level of expertise to confirm that it is of an acceptable scientific standard.

# Peer review: Referee response for version 2

**DOI:** 10.5256/f1000research.4325.r4759

**Published:** 2014-05-13

**Authors:** Thomas Brozoski

**Affiliations:** 1 Division of Otolaryngology, Southern Illinois University, Springfield, IL, USA

I have read this submission. I believe that I have an appropriate level of expertise to confirm that it is of an acceptable scientific standard.

# Peer review: Referee response for version 1

**DOI:** 10.5256/f1000research.3848.r3906

**Published:** 2014-04-22

**Authors:** Ramesh Rajan

**Affiliations:** 1 Department of Physiology, Monash University, Sydney, Australia

This is an interesting area of study and a timely one. I have only a couple of comments to make the article clearer:

1. *“Central neural changes following hearing loss have been described using many different animal models and include changes in tonotopic maps, increased synchronous firing patterns and increased spontaneous firing rates"*  This statement focuses on the “positive” side effects of cochlear hearing loss, but there are many “negative” changes as well such as reduced sensitivity, loss of inhibition (positive and negative used in the same sense as in schizophrenia, not to imply good or bad).
2. The first paragraph of the Introduction is couched in terms of tinnitus, but this article has nothing to do with tinnitus since the authors have examined changes after loud sound but not measured tinnitus, and the link in the Introduction to tinnitus is reported in one brief line. This is not a compelling argument. The demonstration that neural activity increases in the paraflocculus in cases of some behavioural evidence of tinnitus is not an argument that the cerebellum is involved in the generation of tinnitus, as is implied in the first sentence of the paragraph “ *other brain regions are likely to be involved in giving rise to the eventual phantom auditory perception.*” The evidence that the authors cite in the rest of the paragraph shows that the paraflocculus gets auditory input, not that it modulates auditory input or percepts as needed for the argument that paraflocculus activity is linked to tinnitus. More compelling is the more recent Brozoski et al. (2013) article which reports that blockade of NMDA receptor activity in the paraflocculus reduces tinnitus, but the authors don’t cite this.
3. Finally, it is unclear to me how many animals contributed the various data. The authors report n=4 per group in the Methods, but the Table presents data for 4 shams and 2 mechanical trauma animals, and Figure 2 does not cite the n for each group.

I have read this submission. I believe that I have an appropriate level of expertise to confirm that it is of an acceptable scientific standard.

# Response

**Published:** 2014-04-23

**Authors:** wilhelmina Mulders

**Affiliations:** University of Western Australia, Australia

1. The reviewer is quite correct to point out that our list of changes is not comprehensive.
2. The reviewer is correct that we cannot make a direct link between our findings and tinnitus. However, in the Introduction we cite two articles by Brozowski and co-workers (paraflocculus ablation and MEMRI) that do directly implicate the paraflocculus in the modulation of tinnitus. We are grateful for the reviewer pointing out the 2013 article and have included this in the paper.
3. The spreadsheet files with our raw data need to be opened and they show the information the reviewer is requesting. We had 4 animals per group as described in the Methods.

# Peer review: Referee response for version 1

**DOI:** 10.5256/f1000research.3848.r3990

**Published:** 2014-03-06

**Authors:** Thomas Brozoski

**Affiliations:** 1 Division of Otolaryngology, Southern Illinois University, Springfield, IL, USA

The title and abstract are appropriate.

Overall, the methods, results, and conclusions appear to be sound. The study contributes to our evolving understanding of the central nervous system consequences of hearing loss. The results also temper the general hypothesis that loss of peripheral hearing sensitivity leads to a loss of inhibition. Unfortunately, the actual story may be more complex than that.

**Specific Comments:**

- There is a typographical error in the methods paragraph describing gene expression analysis (missing parenthesis).
- Hearing threshold data are displayed as dB loss. This is okay, but I prefer to see hearing threshold levels simply plotted as dB SPL. Elevations (loss) can then be interpreted by the reader.
- It is surprising that GABA indictors were elevated in the trauma animals, while glutamatergic indicators were not. But that is why research is done.
- It would have been informative to look at longer-term consequences of the trauma as well, and perhaps those experiments are in progress.

I have read this submission. I believe that I have an appropriate level of expertise to confirm that it is of an acceptable scientific standard.

# Response

**Published:** 2014-04-23

**Authors:** wilhelmina Mulders

**Affiliations:** University of Western Australia, Australia

1. We will add the missing parenthesis.
2. Our spreadsheet with the raw data shows the hearing thresholds for each animal.
3. We agree that the lack of change in glutamatergic indicators is surprising, especially in view of the reviewer's own result using NMDA antagonist. However, as we point out in our Discussion, our data was derived at an early time-point after trauma. In addition, it is possible that a glutamatergic pathway is involved but not necessarily upregulated in this circuitry.
